# Supplementary material for: Are carbon emissions trading and green financial instruments synergistic? -Comprehensive quantitative research based on content analysis
Source: PLoS One. 2024 Mar 7;19(3):e0298601. doi: 10.1371/journal.pone.0298601 (PMC10919601; doi:10.1371/journal.pone.0298601)
Supplement: S1 Data — (ZIP) [file pone.0298601.s001.zip › Data/地级市Z_F工作报告.zip!/║╙─╧/╨φ▓2╩╨/└┤╫╘╣1⁄2╓┌║┼í╛┬φ┐╦╩2╛▌═°í┐_2019.docx]

各位代表：

现在，我代表市人民政府，向大会报告工作，请予审议，并请各位政协委员和其他列席人士提出意见。

一、2018年工作回顾

2018年是全面贯彻党的十九大精神开局之年。一年来，面对严峻复杂的国际国内形势和艰巨繁重的改革发展任务，在省委、省政府和市委的坚强领导下，全市人民以习近平新时代中国特色社会主义思想为指导，全面落实党的十九大和习近平总书记视察指导河南时的重要讲话精神，坚持稳中求进工作总基调，以新发展理念为引领，以高质量发展为方向，以供给侧结构性改革为主线，深入开展“三大攻坚战”，持续打好“四张牌”，扎实做好“六稳”，统筹稳增长、促改革、调结构、惠民生、防风险，较好完成了经济社会发展主要目标任务。

（一）抓关键稳运行，经济保持平稳增长。全年完成生产总值2830.6亿元、增长8.6%，总量和增速分居全省第4位、第2位；一般公共预算收入166.1亿元、增长14.4%，税收占比超过70%。突出稳定工业增长。强化监测分析，加强运行调节，规模以上工业增加值增长8.5%，高于全国、全省2.3个和1.3个百分点，工业竞争力居全省第3位。着力抓好项目建设。190个重点项目完成年度投资1206亿元，全社会固定资产投资增长10.2%。襄城县硅材料产业园二期、远东传动轴专用车智能化生产线等79个项目开工建设，开普检测公共服务平台等36个项目竣工投产，形成了一批新的经济增长点。万达广场等商业项目建成投用，社会消费品零售总额873.8亿元、增长10.1%。大力发展民营经济。新设立企业1.2万户，市场主体达27万户。森源集团入选“中国企业500强”，4家企业入围“中国民营企业500强”，数量居全省第1位，全国促进民间投资经验交流现场会在许昌召开。强化企业纾困解难。深入贯彻落实习近平总书记在民营企业座谈会上的重要讲话精神，扎实开展“四个一百”专项行动，坚持“解难题、增信心、促发展、建机制”，持续强化企业服务，解决问题1099个，推进380家企业与银行机构开展对接，到位资金74.7亿元，化解66家企业担保链风险，一大批企业渡过难关，促进经济逆势回升，得到省委、省政府充分肯定和广大企业家真诚拥护，“四个一百”已经成为许昌服务企业、优化营商环境的代名词。

（二）补短板强弱项，三大攻坚战扎实推进。防范化解重大风险。稳妥有序化解政府债务风险，坚持“堵后门、开正门”，地方债务风险总体可控。有效防范化解金融风险，去杠杆、治乱象、抓规范、强监管，依法严厉打击非法集资，守住了不发生区域性系统性金融风险的底线。着力化解社会风险，深入开展扫黑除恶专项斗争，打掉涉黑组织4个、黑恶势力犯罪集团（团伙）35个，荣获“全省综治和平安建设优秀省辖市”称号，公众安全感指数、执法满意度分居全省第2位、第3位。依法防范化解非法集资、问题楼盘、信访积案“三类突出问题”，深化重点行业领域治理整顿，社会大局保持和谐稳定。全力推进精准脱贫。聚焦提高脱贫质量，突出精准方略，严格落实“两不愁三保障”标准，全年脱贫1.79万人，9个贫困村实现退出。深入实施稳定增收计划，扎实推进“千企帮千村”，建成扶贫就业基地381个。加强金融扶贫，新增扶贫贷款2亿元，覆盖2.19万贫困户。统筹抓好与淅川县结对帮扶工作，援建项目67个。深入开展污染防治。完成省定工业重点治理项目111个，集中整治“散乱污”企业1110家。全力做好中央环境保护督察“回头看”反馈问题整改工作，交办案件全部办结。严格落实河湖长制，扎实推进“四水同治”，集中式饮用水水源地水质达标率达到100%，连续4年在全省实行最严格水资源管理制度考核中位列第一。全面落实土壤污染防治行动计划，土壤环境质量总体保持稳定。

（三）强基础提质量，“四张牌”打出新成效。加快产业结构优化升级。坚持优化存量和扩大增量“两手抓”，190个产业转型攻坚重点项目完成投资931亿元，265个“三大改造”项目完成投资420亿元，在全省智能制造观摩点评活动中荣获小组第一。抢抓5G产业发展机遇，网络与交换技术国家重点实验室（北邮）许昌基地揭牌成立，全国首个5G泛在小镇项目正式启动，成功举办中国·许昌5G泛在峰会。大力发展现代服务业，实现服务业增加值1047.4亿元，占生产总值的比重达37%。现代金融业稳健发展，与光大银行、兴业银行签订战略合作协议，获得授信600亿元，恒达集团在香港成功上市。健康养老产业加快发展，入选全国首批城企联动普惠养老专项行动试点城市，国家健康养老示范区、国家森林康养基地规划编制完成，鄢陵县成为全国智慧健康养老示范基地。强化创新驱动发展。“许昌英才计划”扎实推进，新建院士工作站2家、省级工程技术研究中心12家，新认定创新创业人才（团队）26个、高层次人才341名，新增高新技术企业47家，新认定国家科技型中小企业181家，1个项目入选国家科技进步二等奖，15个项目入选省科技进步奖。新增专利申请量1.1万件、授权量6300余件，均居全省第4位。百名科学家许昌行活动成果丰硕，国家高新区创建通过科技部评审。提升基础能力建设。实施交通基础设施项目48个，新元大道东段拓宽提质工程建成通车，国道107线许昌境改建工程具备通车条件。重点水利工程顺利实施，引黄入长济许工程实现通水。国土绿化全面提速，完成营造林30万亩，其中国家储备林13万亩。城市供热能力不断增强，市区集中供热普及率超过75%，供热期由3个月延长到4个月。大力推进新型城镇化。常住人口城镇化率达到52.63%。郑许一体化迈出坚实步伐，郑许一体化发展规划、许港产业带空间规划编制完成，郑许市域铁路全面开工，郑万、郑合铁路许昌段和4个高铁站建设顺利。百城建设提质工程扎实推进，395个项目完成投资564亿元，职教园区等一批项目建成投用。棚户区改造新开工2.21万套，基本建成2.66万套，超额完成省定目标。深入实施“四改一增”，改造老庭院119个、老街道34条、老厂院9个、老市场4个，增加停车位近2000个。

（四）重统筹促融合，乡村振兴加快实施。突出规划引领。在全省率先出台关于推进乡村振兴战略的实施意见，高标准编制完成乡村振兴战略规划。稳定粮食生产能力。全面落实“藏粮于地、藏粮于技”战略，新建高标准粮田10.9万亩、高效节水灌溉面积18.4万亩，粮食产量达到298万吨，创历史新高。持续深化农业供给侧结构性改革，加快推进“四优四化”，优质强筋小麦种植面积达到60万亩。新增新型农业经营主体1000余家，省级农业产业化集群达到10个。加强美丽乡村建设。实施改善农村人居环境三年行动计划，改善农村人居环境工作连续两年居全省第1位。深化与中民投合作，积极探索乡村振兴“政企合作”新模式，首批中民乡邻广场项目顺利落地。强化农村公共服务。新建改造“四好农村路”400公里，新增农村自来水供水人口47.2万人，新建农村4G基站290个，近一半村庄建成卫生公厕。建设基层综合性文化服务中心2400余个，基本实现乡村公共文化服务全覆盖。完善乡村治理体系，全面推进移风易俗，文明村镇率接近30%。

（五）抓改革扩开放，发展活力不断增强。纵深推进重点领域改革。深化“放管服”改革，“一网通办”前提下的“最多跑一次”改革全面提速，投资项目审批“容缺办理”模式全面推广，审批服务更加便民化，行政审批事项保持全省最少，“一窗受理、集中服务”等工作走在全省前列。深化商事制度改革，企业开办时间由6个工作日压缩到3个工作日以内，迈进全国先进行列。深化国企改革，市属11家国有“僵尸企业”处置工作提前完成。深入实施质量强市战略，成为全国唯一地级市标准化改革创新先行区创建单位。有序推进机构改革，机构设置和职能配置得到优化。财税、金融、农业农村、综合执法等领域改革稳步推进。不断提升对外开放水平。坚持每季度在发达地区举办专题招商活动，积极承接产业转移，成功举办三国文化旅游周、花博会、药交会，新增省外资金项目313个，实际到位省外资金508.7亿元。主动融入“一带一路”建设，与德国、东盟等国家和地区的合作不断深化，成功举办2018中德智能制造合作主题论坛，获批全省首个中德（许昌）中小企业合作区，对德合作签约项目23个、落地10个，达成合作意向27个。加强开放平台建设，许昌海关监管查验中心建成运营，经济技术开发区国际合作产业园获批建设。

（六）稳就业惠民生，人民群众获得感持续提升。财政民生支出227.1亿元，占一般公共预算支出的71.3%，省、市民生实事圆满完成，城市宜居度、幸福感、获得感、居民对生态环境的满意度4项指数均居全省第1位。持续扩大社会就业。新增城镇就业7.3万人、农村劳动力转移就业5.2万人，城镇登记失业率2.99%，居民人均可支配收入达23821元、增长9.2%。稳步提高社会保障水平。五项社会保险基本实现法定人群全覆盖，企业和机关事业单位退休人员基本养老金年人均分别提高1552元和1595元，城乡居民基础养老金最低标准年人均提高216元。全面发展社会事业。深入实施基础教育提升三年攻坚计划，中心城区新建改扩建中小学10所、幼儿园20所，新增学位1.6万个。健康城市创建扎实推进，医药卫生体制改革持续深化，市中心医院新院区加快建设，市妇幼保健院新院区即将投用，成功举办市八运会。新建智慧阅读空间18座，“15分钟”医疗服务圈、养老服务圈、健身圈、阅读圈、便民服务圈更加完善。强化食品安全监管，顺利通过国家食品安全示范城市中期评估，食品安全满意度居全省第1位。

积极推进军民融合发展，加强人民防空建设，河南省（许昌）军民融合产业基地获批，驻许人民解放军、武警部队、预备役人员、民兵为地方发展作出新贡献，双拥工作持续深化。妇女儿童、民族宗教、残疾人、红十字、慈善等事业健康发展。外事、侨务、港澳、对台、统计、史志、气象、地震、援疆等工作取得新成绩。

与此同时，市政府高度重视自身建设，深入学习贯彻习近平新时代中国特色社会主义思想，树牢“四个意识”，坚定“四个自信”，坚决做到“两个维护”，自觉在思想上政治上行动上同以习近平同志为核心的党中央保持高度一致。严格落实全面从严治党要求，全面加强政府系统党的建设，抓好中央、省委巡视反馈问题整改，坚决贯彻中央、省决策部署和市委工作要求。自觉接受人大依法监督、政协民主监督和社会舆论监督，虚心听取各民主党派、工商联、无党派人士意见、建议，91件人大代表建议（议案）、199件政协提案全部办结。加强法治政府建设，推进依法行政，强化审计监督，持续纠正“四风”，政府自身建设和治理能力不断提升，风清气正、干事创业的良好氛围更加浓厚。

各位代表！

过去一年我们取得的发展成绩，是以习近平同志为核心的党中央掌舵领航的结果，是省委、省政府和市委坚强领导的结果，是全市上下团结一心、开拓奋进的结果。在此，我代表市人民政府，向全市人民，向驻许人民解放军指战员、武警官兵、公安民警，向各民主党派、工商联、各人民团体致以崇高的敬意！向所有关心支持许昌发展的社会各界人士表示衷心的感谢！

在看到成绩的同时，我们也清醒认识到，我市经济社会发展还存在不少困难和问题。一是发展质量仍需提升。与先进地区相比，我市新旧动能接续转换较慢，结构性矛盾依然突出，创新能力不强，城乡发展仍不平衡，转型发展任务还很艰巨。二是实体经济困难较多。经济下行压力加大，市场需求增长放缓，用工、用地等成本较高，融资难、融资贵问题普遍存在，营商环境仍需持续优化。三是潜在风险依然突出。资源环境约束加剧，企业风险、金融风险、债务风险不容忽视，安全生产形势仍不容乐观。四是民生领域存在短板。教育、文化、卫生、养老等社会事业和污染防治距群众期盼还有一定差距。同时，政府职能转变还不到位，服务效能还不够高，个别地方和领域不正之风和腐败问题仍有发生。对上述问题，我们一定高度重视，在今后的工作中认真加以解决。

二、2019年目标任务

2019年是中华人民共和国成立70周年，是全面建成小康社会、实现第一个百年奋斗目标的关键之年。习近平新时代中国特色社会主义思想为我们指明了前进方向，省委、省政府对我们提出了明确要求。省委书记王国生同志指出，“许昌要在践行新发展理念上为全省探索路径、打造样板”；省长陈润儿同志提出，“许昌要在推进产业转型升级、加快县域经济发展、推动改革开放创新上奋力走在全省前列”。这既是殷切期望，也是光荣使命，更是难得机遇。我们必须全心全意、尽心尽力抓好贯彻落实。

“探索路径、打造样板、走在前列”，我们必须坚定信心决心，切实增强使命感。近年来，许昌经济社会发展始终保持良好态势，高质量发展迈出坚实步伐，城市知名度、美誉度和竞争力持续攀升，得到了省委、省政府和社会各界充分肯定。新时代呼唤新使命，新使命开启新征程。许昌已经站在了更高的起点上，在全省全国大局中的地位更加凸显、作用更加重要。面对省委、省政府的新要求、新定位，许昌如何探索路径、打造什么样的样板、怎样实现“三个走在前列”，加快新发展理念之路由“像”到“是”转变，是摆在我们面前的一道时代“必答题”。为此，我们必须树立崇高的历史使命感，坚持以习近平新时代中国特色社会主义思想为指导，坚持以人民为中心的发展思想，认真落实省委、省政府决策部署，为中原更加出彩作出新的更大贡献。

“探索路径、打造样板、走在前列”，我们必须保持清醒忧患，切实增强危机感。今年经济发展面临的环境更加复杂严峻，世界经济下行压力逐步加大，中美贸易摩擦带来的经济不确定性增加，市场需求放缓，增长动能减弱，经济运行稳中有变、变中有忧，稳定经济增长的任务更加繁重。环顾周边，城市竞争日趋激烈，各地都在你追我赶、竞相发展，我市正处在“不进则退、慢进亦退”的重要关口。为此，我们必须保持如履薄冰的危机感，全面对标先进，查找自身不足，进一步解放思想，理清思路，自加压力，再添动力，当好全省高质量发展的排头兵。

“探索路径、打造样板、走在前列”，我们必须抢抓发展机遇，切实增强紧迫感。事在人为，危中有机，抓住机遇才能赢得发展。从国家看，中央继续实施积极的财政政策和稳健的货币政策，加快实施一批补短板、增后劲的重点项目，围绕实现“六稳”打出了加快发展政策“组合拳”。从全省看，“五区联动”“四路协同”效应进一步显现，中原城市群实力持续增强，“三大改造”、百城建设提质等重大项目和工程扎实推进。从我市看，经济运行总体平稳的态势没有改变，产业基础更加雄厚，营商环境持续优化，新的经济增长点不断涌现，特别是郑许一体化深入推进，为我市高质量发展带来了更为广阔的空间、更大的潜力。为此，我们必须增强坐不住、等不起、慢不得的紧迫感，既要醒得早，更要起得快，扬长补短，抢占先机，牢牢把握发展主动权，加快打造“智造之都、宜居之城”。

“探索路径、打造样板、走在前列”，我们必须树牢担当意识，切实增强责任感。担当既是改革的需求，也是人民的期盼。当前，我们正处于“船到中流浪更急、人到半山路更陡”的关键时期，肩负着全市498万人民过上更加美好幸福生活的重大历史责任，容不得须臾放松和丝毫懈怠。为此，我们必须坚定舍我其谁的责任感，勇于担当、直面矛盾、解决问题、积极作为，只要有利于发展、有利于人民幸福，就大胆闯、敢于干，努力创造出不负时代的光辉业绩。

今年政府工作要认真落实市委七届八次全会要求：以习近平新时代中国特色社会主义思想为指导，全面贯彻党的十九大和十九届二中、三中全会精神及中央经济工作会议精神，认真落实省委十届八次全会精神，统筹推进“五位一体”总体布局，协调推进“四个全面”战略布局，坚持稳中求进工作总基调，坚持新发展理念，坚持推动高质量发展，坚持以供给侧结构性改革为主线，坚持深化市场化改革、扩大高水平开放，继续打好三大攻坚战，持续推进“四个着力”、打好“四张牌”，加快推进郑许一体化，大力抓好“四个一百”专项行动，统筹推进稳增长、促改革、调结构、惠民生、防风险、保稳定工作，进一步稳就业、稳金融、稳外贸、稳外资、稳投资、稳预期，切实增强人民群众获得感、幸福感、安全感，保持经济持续健康发展和社会大局稳定，为全面建成小康社会收官打下决定性基础，在践行新发展理念上为全省探索路径、打造样板，在中原更加出彩中走在前列，以优异成绩庆祝中华人民共和国成立70周年。

今年经济社会发展主要预期目标是：生产总值增长7.5%左右，全员劳动生产率增长高于全省平均水平，一般公共预算收入增长8%左右，居民消费价格指数涨幅控制在3%左右，经济结构持续优化，质量效益明显提升。创新发展取得新成果，研究与试验发展经费投入强度达到1.94%，战略性新兴产业增加值占规模以上工业比重达到20%以上。协调发展取得新成效，常住人口城镇化率提高1.5个百分点左右，人均预期寿命持续提高，九年义务教育巩固率达到94.5%，医疗卫生、文化、体育等社会事业全面进步。绿色发展取得新提升，单位生产总值能耗下降、主要污染物排放量实现省下达目标。开放发展取得新突破，进出口保持平稳增长，营商环境进一步优化。共享发展取得新进步，居民人均可支配收入增长高于经济增长，城镇登记失业率控制在4%以内。以上目标，更加凸显了新发展理念和高质量发展要求，有利于把工作重点放在推进高质量发展上来。

三、2019年重点工作

今年，要围绕省委、省政府“探索路径、打造样板、走在前列”的要求，突出重点、把握关键，扎实做好9 个方面的工作。

（一）着力稳定经济增长，夯实高质量发展基础。坚定不移支持实体经济发展，充分发挥有效投资的关键作用和消费的基础作用，科学施策，精准出招，保持经济稳中向好态势。

深入实施“四个一百”专项行动。认真贯彻习近平总书记在民营企业座谈会上的重要讲话精神，按照“解难题、增信心、促发展、建机制”总要求，着力解决困扰企业发展的具体问题。进一步扩大“四个一百”覆盖范围，加强企业纾困帮扶工作。坚持“政府办好围墙外的事，企业办好围墙内的事”，全力优化营商环境，营造公平、透明、法治的发展环境，提振民间投资信心。持续构建亲清新型政商关系，营造有利于企业家健康成长的良好氛围，畅通企业家投诉渠道，依法保护其合法权益，让企业家安心搞经营、放心办企业。提高金融服务实体经济能力，强化银企对接，鼓励金融机构优化信贷结构、扩大信贷投放、降低贷款成本，打通金融服务实体经济的“血脉”。大力实施大企业集团培育计划和企业家队伍培养“十百千”行动计划，在市场竞争中打造一支有开拓精神、前瞻眼光、国际视野的企业家队伍，推动企业做强做大，新增主板上市企业1家以上。落实中央更大规模减税降费政策，切实让市场主体特别是小微企业有明显减税降费感受，坚决兑现对企业和社会的承诺。

切实提高投资质量。聚焦产业转型升级、城市功能配套、生态环境建设、交通水利基础设施等重点领域和薄弱环节，大力实施投资促进计划，确保全市171个重点项目完成年度投资1000亿元以上。用好政府投资杠杆撬动作用，鼓励社会力量进入教育、文化、医疗、健康养老等领域，充分激发民间投资活力。

积极促进消费升级。抢抓国家促进形成强大国内市场的机遇，挖掘健康养老、育幼、休闲、信息、旅游等领域和传统服务业消费潜力，培育家政服务、现代生活用品等新的消费热点，落实个人所得税专项附加扣除政策，增强消费能力。加快发展农村电商，开拓农村消费市场。不断拓宽居民劳动收入和财产性收入渠道，促进实物消费提档升级，加强市场监管和消费者权益保护，让群众“能消费”“愿消费”“敢消费”。

（二）着力打好打赢三大攻坚战，坚守高质量发展底线。强化问题导向，坚持底线思维，把三大攻坚战摆在更加突出的位置，全力攻坚，务求实效。

防范化解重大风险。认真落实习近平总书记在省部级研讨班上重要讲话精神，研究应对和化解风险挑战的有效措施，既打好防范和抵御风险的有准备之战，也打好化险为夷、转危为机的战略主动战。依法规范政府举债行为，认真落实“八个一批”解决方案，坚持“堵后门、开正门”，加大专项债券申报力度，化解存量，遏制增量，有效防范化解地方债务风险。全面加强金融监管，积极防控非法集资、企业信用违约、股票质押平仓、融资担保链、互联网借贷等领域风险隐患，高度关注房地产领域金融风险，开展金融生态县（市）考核评价，持续优化金融生态环境，守住不发生区域性系统性金融风险的底线。有效解决中小微企业融资难、融资贵问题，探索开展民营企业债转股，引导企业加大股权融资力度，降低企业经营风险。

推进精准脱贫。坚持精准方略，把提高脱贫质量放在首位，确保工作务实、过程扎实、结果真实。聚焦99个重点村和因病因残致贫群体，重点解决实现“两不愁三保障”面临的突出问题，完成1.66万贫困人口稳定脱贫任务，做好社会保障兜底贫困人口的基础设施建设，关注非贫困村的建设和发展。深入实施稳定增收计划，扎实开展“千企帮千村”活动，因地制宜发展扶贫产业，实现对有劳动能力和发展意愿的贫困人口产业帮扶全覆盖，切实减少和防止贫困人口返贫。统筹抓好我市与淅川县、长葛市与桐柏县结对帮扶工作。

抓好污染防治。扎实开展污染防治攻坚战三年行动，认真整改中央环境保护督察“回头看”反馈问题，深入实施经济结构提质、生态功能提升、国土绿化提速、环境治理提效“四大行动”，加快城市通风廊道规划建设，推动环境质量持续提升、空气质量明显改善，给人民群众更多碧水蓝天。统筹推进城乡污水处理能力建设，严格落实河湖长制，全面落实“四水同治”，加强饮用水水源地保护，逐步消除城乡黑臭水体。严格保护未污染土壤，推进受污染耕地安全利用和治理修复，确保粮食和人居环境安全。

（三）着力推进郑许一体化，抓牢高质量发展“龙头”。坚持立足许昌、对接空港、发挥优势、错位发展，持续加强交通、产业、生态、平台、功能、空间对接，形成“1+1>2”的协同发展效应。

强化交通对接。高标准推进郑许市域铁路建设，完成年度投资35亿元。统筹谋划沿线站点与周边交通横向衔接，加强周边区域规划管控、产业布局、功能构建、人口集聚，带动沿线经济社会发展。谋划建设大型综合性陆港交通枢纽，无缝衔接“四条丝绸之路”。抓好郑万、郑合高铁许昌段及4个高铁站建设。加快忠武路规划建设，推进国道230线、省道227线许昌境和文峰北路提升改造，构建郑许立体快捷综合交通体系。

强化产业对接。以打造许港产业带为重点，突出抓好5G泛在小镇、许昌中德（欧）产业园和长葛产业新城等产业项目建设，积极承接郑州航空港、河南自贸区产业辐射和溢出，吸引更多优质产业和企业入驻。积极争取国家战略性新兴产业集群布局，着力打造大都市区先进制造业基地、区域现代物流中心、临空产业基地。

强化生态对接。围绕生态功能区定位，加快郑许生态廊道绿化、中华生物园、双洎河国家湿地公园、中心城区生态园林城市提升等生态项目建设，加强清潩河长葛段综合治理，打造独具魅力的郑州大都市区“南花园”。

强化平台对接。积极申建中国（河南）自由贸易试验区许昌联动发展区和保税物流中心（B型），不断完善提升跨境电商查验中心、跨境电商基地等开放平台，探索区域协调发展新路径。

（四）着力加快转型升级，增强高质量发展动能。坚持以产业高端化、技术尖端化、质量顶端化为目标，一手抓优化存量，一手抓扩大增量，加快建设现代产业体系。

大力发展先进制造业。开展“智能制造引领、重点技术攻坚、传统产业提质、新兴产业培育”四大行动，加快实施森源电气产业基地三期、平煤隆基高效单晶硅电池片二期等一批工业重点项目，培育许昌智慧信息、禹州绿色铸造、许昌远东传动轴智能制造等一大批产业园区，重点支持主营业务收入100亿元以上大型制造业企业和10亿元以上高成长制造业企业发展。深入实施“三大改造”，重点推进智能电力装备、新能源及网联汽车、高纯硅材料、5G等九大产业发展专项，抓好总投资1934亿元的211个重点产业项目，积极培育新的经济增长点，力争通过3到5年努力，打造6个千亿级产业集群。全面落实质量强市战略，大力开展质量提升行动，推动标准与国际先进水平对接，提升产品和服务品质，加快培育国际、国内竞争新优势。

加快提升现代服务业。抓住全国首批城企联动普惠养老专项行动试点城市建设机遇，积极发展健康养老产业，加快“一院一校一中心”、康养小镇等项目建设，大力发展医养结合，提升居家、社区和机构养老服务水平，争创国家健康养老示范区。大力发展冷链物流、快递物流、电商物流，努力引进国内外知名物流企业。大力发展全域旅游，推进曹魏古城、神垕古镇等项目建设。加快发展普惠金融、科技服务、文化创意等产业，力争引进银行业金融机构、保险机构各1家。积极培育专业服务、特色消费、文化体验等新兴服务业。

持续强化科技创新。认真落实创新驱动发展战略规划，聚焦培育“四个一批”，积极促进“四个融合”，加快提升创新发展水平。实施创新龙头企业提升引领工程、高新技术企业培育工程和“科技小巨人”企业培育工程，新增高新技术企业30家以上、科技型中小企业50家以上，新建国家级、省级创新平台10个以上，市级创新平台30个以上，实施转型升级创新专项10个以上，推动国家高新区尽快获批。深入实施“许昌英才计划”，坚持引进外部人才和培育本地人才并重，加快高层次人才服务中心和人才公寓建设，力争新增院士工作站1至2个。

（五）着力深化改革开放，激发高质量发展活力。贯彻落实庆祝改革开放40周年大会精神，加快市场化改革，扩大高水平开放，充分激发经济社会发展动力活力。

深化重点领域改革。深入推进供给侧结构性改革，认真贯彻落实“巩固、增强、提升、畅通”八字方针，确保完成省定目标任务，促进经济提质增效。深入推进“放管服”改革，持续简化审批、优化服务，把“一网通办”前提下的“最多跑一次”改革落到实处，为市场主体添活力，为人民群众增便利。加强“双随机、一公开”监管，优化环保、消防、税务、市场监管等执法方式，以公正监管管出公平、管出效率、管出活力。稳步推进国资国企改革，深化企业产权结构、组织结构、治理结构改革，建立规范的现代企业制度。完善国有资产监管，理顺国有金融资本管理体制机制，加快由管企业向管资本转变。持续深化农村改革，完善农村承包地“三权分置”办法，开展土地经营权入股产业化经营试点，有集体经济收入的村庄达到70%。扎实推进政府机构改革，优化职能配置，提高行政效能，确保按期圆满完成。有序推进政府融资平台改革，深化市建设投资公司体制改革，做大规模、做强实力，力争2020年公司规模达到300亿元。同时，不断深化财税、生态环保体制、城市综合执法、公共资源交易平台整合共享等改革。

推进更高水平开放。深度融入“一带一路”建设，抢抓我省“五区联动”“四路协同”机遇，围绕“三个20”对德合作目标，深化智能制造、节能环保、健康养老等领域合作，打造全省中德合作典范。开展专业化精细化招商，围绕智能制造、数字经济、生物医药、电子信息等重点产业，引进一批实力突出的“旗舰型”企业、竞争力强劲的行业“领头羊”、细分领域“隐形冠军”、高科技“独角兽”落户许昌。更加注重招商实效，高质量办好三国文化旅游周、花博会、鄢陵国际健康峰会、禹州钧瓷文化旅游节等活动，切实提高合同履约率、资金到位率、项目开工率。

（六）着力推动新型城镇化，强化高质量发展支撑。坚持以人的城镇化为核心，以城市高质量发展为目标，着力打造城乡区域协调发展新格局。

大力实施百城建设提质工程。统筹百城建设提质与文明城市创建，着力推进城市规划、建设、管理、经营高质量，促进城市由外延扩张向内涵提质转变。稳步推进市级空间规划试点建设。实施好中心城区140个百城建设提质项目，完成年度投资264亿元。更好解决群众住房问题，落实城市主体责任，促进房地产市场平稳健康发展，有序推进棚户区改造，保障困难群体基本居住需求。稳步推进“四改一增”工程，改造提升老庭院64个、老厂院12个、老市场9个、老街道30条，增加停车位1000个左右，积极推进加装电梯试点工作，为全国老旧小区改造创造经验。

持续深化城市创建。深化中心城区“五城联创”和县（市）城区“三城联创”，持续巩固创建成果，力争中国人居环境奖拿杯夺牌。突出抓好交通秩序“治堵”、市容卫生“治脏”、生态环境“治污”、公共服务“治差”，提高柔性化治理、精细化服务水平，让许昌更加宜居，更具包容和人文关怀。

强化基础能力建设。加大城际交通、物流、市政基础设施等建设力度，加快推进许信高速、国道311线许昌境改建等重大交通项目，加强运输场站和交通物流园区建设，完善公路运输和服务网。完善水利基础设施，加快实施中心城区河湖水系水源开发、颍汝干渠综合整治等重大水利专项。推进供热能力建设，新建改建热力管网14.2公里，新增集中供暖面积150万平方米。

积极推动农业转移人口市民化。统筹户籍制度改革和城镇基本公共服务均等化，认真落实“1+6”配套政策体系，实行进城落户政策“零门槛”，抓好已经在城镇就业的农业转移人口落户工作，让农业转移人口“进得来、留得住、过得好”。

（七）着力实施乡村振兴战略，补齐高质量发展短板。认真贯彻习近平总书记在参加十三届全国人大二次会议河南团审议时的重要讲话精神，按照乡村振兴战略总要求，围绕实现农业强、农村美、农民富，把“三起来”“三结合”落到实处，加快推进产业、人才、文化、生态、组织“五个振兴”。

扛稳粮食安全重任。推动藏粮于地、藏粮于技，稳步提升粮食产能，新建和提升高标准粮田15万亩，新增高效节水灌溉面积15万亩，粮食种植面积稳定在650万亩左右，总产稳定在280万吨以上。全面巩固“大棚房”问题整治成果，牢牢守住耕地保护红线。扎实推进“四优四化”，优质专用小麦发展到80万亩。

发展现代农业产业。坚持以“粮头食尾”“农头工尾”为抓手，深入推进农业供给侧结构性改革，延伸产业链、提升价值链、打造供应链。实施农产品加工业提升行动，扶优扶强农业产业化龙头企业，市级以上龙头企业稳定在100家以上，主要农产品加工转化率达到70%。发展多种形式农业适度规模经营，积极培育农民专业合作社、家庭农场，新增示范性新型农业经营主体100家。

整治农村人居环境。学习浙江“千万工程”经验，扎实推进农村人居环境三年整治行动，推动生产、生活、生态协调发展。补齐农村基础设施短板，加快推进“厕所革命”，新建农村道路150公里、通信基站1170个，新增农村自来水受益人口10.46万，自来水普及率达到95%以上，完成15万户省定户厕改造任务。深化与中民投公司合作，加快美丽乡村文化综合体项目建设。

提高乡村治理效能。深入开展农村精神文明创建活动，大力推动移风易俗，保护好农村传统文化、乡土文化，培育文明乡风、良好家风、淳朴民风。加强农村基层组织建设，深化拓展“四议两公开”，发挥农民主体作用，构建自治、法治、德治相结合的乡村治理体系，打造充满活力、和谐有序的善治乡村。

（八）着力加强生态文明建设，厚植高质量发展优势。深入贯彻习近平生态文明思想，牢固树立和践行“绿水青山就是金山银山”理念，保持加强生态文明建设的战略定力，探索以生态优先、绿色发展为导向的高质量发展之路。

扎实推进国家生态文明先行示范区建设。坚持生态惠民、生态利民、生态为民，深入开展山水林田湖草一体化生态保护和修复，统筹推进森林、湿地、流域、农田、城市五大生态系统建设，探索建立资源环境生态红线管控制度及资源环境承载能力监测预警机制。健全完善秸秆、建筑垃圾综合利用及城市矿产回收利用的许昌模式，力争在生态文明制度创新上取得新突破。

加快国土绿化提速行动。树立大绿化、大生态理念，主动融入森林河南建设，大力实施“绿满许昌”行动计划，推进山区森林化、平原林网化、城市园林化、乡村林果化、廊道林荫化和庭院花园化，构建“点、线、面、网、带”相结合的森林生态体系，完成28.28万亩林业生态建设任务。推动花木产业壮大规模、提档升级，让“中国花木之都”名片更加闪亮。加强山区、湿地生态修复和保护，规划建设一批湿地公园、城郊森林公园、森林乡村，把好山好水好风光融入城市。

大力发展循环经济。开展节能减排技术改造和节能降耗行动，推进清洁生产，推动绿色循环低碳发展。争创“无废城市”建设试点，持续推进固体废物源头减量和资源化利用，着力形成固体废物环境影响降至最低的城市发展模式。支持企业开展节水、节能、节材、节地生产技改活动，大力淘汰落后产能，形成资源节约集约运行机制。加快静脉产业园、襄城县循环经济产业集聚区、许昌（魏都）循环经济产业园建设，支持鄢陵县省级低碳试点城市创建。

（九）着力保障和改善民生，共享高质量发展成果。坚持以人民为中心，尽力而为、量力而行，全力办好省定、市定民生实事，不断提升人民群众的获得感、幸福感、安全感。

稳定扩大就业创业。把稳就业摆在更加突出位置，落实就业优先政策，加强对灵活就业、新就业形态的支持。持续实施全民技能振兴工程，开展各类职业技能培训10万人次。实施农民工返乡就业创业专项行动，抓好高校毕业生、退役军人、农民工等重点群体就业，加强对城镇各类就业困难人员的就业帮扶，实现城镇新增就业5万人、农村劳动力转移就业2万人。

加快教育改革创新。打好基础教育提升三年攻坚计划收官战，圆满完成新建、改扩建200所中小学、100所幼儿园任务。深入推进教育“1+1+6”综合改革，加快推进校长职级制、教师“县管校聘”等改革，逐步建立现代教育制度体系，持续提升教育质量。加强教师队伍建设，改善教师工作生活条件，让教师成为最受尊崇的职业。强化综合治理，维护校园安全，健全学生资助制度，使更多孩子享受到更加公平更有质量的教育。

提升社会保障水平。强化兜底保障功能，大力实施全民参保计划，实现法定人员全覆盖。继续提高城乡居民基础养老金最低标准，调增企业和机关事业单位退休人员基本养老金。推进基本医疗保险支付方式改革，提升基本医保、大病保险、困难群众大病补充医疗保险保障水平。提高城乡低保、特困供养、孤儿养育等保障标准，加强残疾人关爱、社会救助、社会福利、优抚安置工作。

加快健康许昌建设。深化公立医院改革和县域综合医改，建立现代医院管理制度，加快市中心医院新院区等项目建设，打造区域医疗中心。加强国家中医药综合改革试验区建设，实施中医药传承创新工程。深入推进农村敬老院三年综合提升改造计划。广泛开展全民健身活动，提高人民群众健康水平。深化国家食品安全示范城市创建，加强食品药品安全监管，让群众吃得更放心、用药更安全。

丰富群众精神文化生活。强力推进国家公共文化服务体系示范区创建，深入实施文化惠民工程。积极培育和践行社会主义核心价值观，广泛开展群众性精神文明创建活动，大力弘扬奋斗精神、科学精神、劳模精神、工匠精神，汇聚向上向善的强大合力。加强互联网内容建设，支持媒体融合发展。加强文化遗产保护利用，繁荣文艺创作，丰富人民群众文化生活。

全力维护社会稳定。深入推进扫黑除恶专项斗争，保持对黑恶犯罪的高压严打态势，在打防并举、标本兼治上下功夫，坚决做到除恶务尽，坚决守护好人民群众的平安生活。加快建设突发事件预警信息发布和应急管理指挥系统，加强突发事件预防处置，提高防灾减灾救灾能力。压实安全生产责任，强化重点领域专项治理，坚决遏制重特大事故发生。加强和创新社会治理，学习推广“枫桥经验”，新建规范化城市社区44个，构建城乡社区治理新格局。开展阳光信访、责任信访、法治信访，综合治理房地产领域问题楼盘信访突出问题，持续打好信访矛盾化解攻坚战，促进人民群众合理合法利益诉求及时、就地解决。持续推进普法教育，开展法治宣传教育进校园、进农村、进社区、进机关、进企业、进家庭。扎实做好庆祝中华人民共和国成立70周年安保等工作，维护社会大局和谐稳定。

加强国防教育、国防动员和国防后备力量建设，支持国防和军队改革，支持驻许解放军、武警部队、预备役部队、民兵和人民防空建设，深入开展双拥共建，加强退役军人服务保障体系建设，推进军民融合深度发展。支持工会、共青团、妇联等人民团体更好发挥桥梁纽带作用。做好民族、宗教、外事、侨务、港澳、对台工作。加强统计、史志、档案、气象、地震、邮政、援疆等工作。

四、加强政府自身建设

面对新的形势任务和人民群众的更高期盼，我们必须更加注重自身建设，持续提升治理能力和水平，加快建设人民满意的法治政府、创新政府、廉洁政府和服务型政府。

（一）加强政治建设，始终对党忠诚。树牢“四个意识”，坚定“四个自信”，坚决维护习近平总书记党中央的核心、全党的核心地位，坚决维护党中央权威和集中统一领导，在政治立场、政治方向、政治原则、政治道路上同以习近平同志为核心的党中央保持高度一致。严守政治纪律和政治规矩，听从党中央号令，紧跟党中央步伐，有令则行、有禁则止，不讲条件、不搞变通，确保党中央决策部署不折不扣落地落实。

（二）坚持依法行政，规范权力运行。严格遵守宪法法律，按照法定权限和法定程序行使权力。善于运用法治思维和法治方式深化改革、推动发展、化解矛盾、维护稳定，把政府活动全面纳入法治轨道。推动科学、民主、依法决策，确保政府的每一项决策都遵守宪法法律、体现人民意愿。坚持严格规范公正文明执法，切实维护公平正义。自觉接受人大依法监督和政协民主监督，主动接受社会舆论监督，认真听取各民主党派、工商联、无党派人士和各人民团体意见，做到有权必有责、用权受监督。

（三）全面加强学习，练就过硬本领。深入推进“两学一做”学习教育常态化制度化，扎实开展“不忘初心、牢记使命”主题教育，不断推动学习贯彻习近平新时代中国特色社会主义思想往深里走、往实里走、往心里走。提高政治能力，增强政治敏锐性、政治鉴别力，做到眼睛亮、见事早、行动快。坚持学习常态化，大兴调查研究之风，加快知识更新，持续提升专业能力，大力营造崇尚学习的良好氛围。注重实践锻炼，学以致用、用以促学、学用相长，不断提高破解难题的能力水平。增强斗争意识，发扬斗争精神，讲究斗争艺术，自觉站到斗争前沿，沿着矛盾上，顶着压力冲，不断提升斗争本领。

（四）持续转变作风，勇于担当作为。坚持对发展负责、对历史负责、对人民负责，保持锐意进取、永不懈怠的精神状态和敢闯敢干、一往无前的奋斗姿态。坚决反对和整治一切形式主义、官僚主义，认真贯彻中央关于“基层减负年”重大部署，让干部把精力用在解决实际问题上。大幅度精简文件和会议，少开会、开短会，开管用的会。优化改进各种督查检查考核和调研活动，严格控制总量，强化结果导向，做到轻车简从、务求实效。健全激励约束机制和尽职免责机制，为担当者担当，为负责者负责，保护干部干事创业的积极性。政府及其工作人员要大力弘扬焦裕禄同志“三股劲”，抓落实、敢担当，高效率、快节奏，令行禁止，以推动改革发展的成果说话，以干事创业的实绩交卷。

（五）牢记为民宗旨，保持清廉本色。全心全意为人民服务、真心实意为人民办事，始终与人民群众站在一起、想在一起、干在一起，既“身入”基层，又“心到”群众，想群众之所想、急群众之所急、忧群众之所忧。落实全面从严治党要求，坚决贯彻落实中央八项规定及其实施细则精神，持之以恒纠治“四风”。加强廉洁政府建设，深入推进政府系统党风廉政建设和反腐败工作，严格遵守廉洁自律准则，一体推进不敢腐、不能腐、不想腐，坚决铲除滋生腐败的土壤。

各位代表！

奋斗创造幸福，实干成就梦想。让我们在省委、省政府和市委的坚强领导下，更加紧密地团结在以习近平同志为核心的党中央周围，高举习近平新时代中国特色社会主义思想伟大旗帜，坚定信心，真抓实干，勇往直前，在谱写新时代中原更加出彩的绚丽篇章中走在前列，以优异成绩迎接中华人民共和国成立70周年！
